# Ambient Air Temperature Does Not Predict whether Small or Large Workers Forage in Bumble Bees (*Bombus impatiens*)

**DOI:** 10.1155/2010/536430

**Published:** 2010

**Authors:** Margaret J. Couvillon, Ginny Fitzpatrick, Anna Dornhaus

**Affiliations:** 1 Department of Ecology and Evolutionary Biology, University of Arizona, Tucson, AZ 85721, USA; 2 Laboratory of Apiculture and Social Insects, Department of Biological and Environmental Science, University of Sussex, Falmer, Brighton BN1 9QG, UK

## Abstract

Bumble bees are important pollinators of crops and other plants. However, many aspects of their basic biology remain relatively unexplored. For example, one important and unusual natural history feature in bumble bees is the massive size variation seen between workers of the same nest. This size polymorphism may be an adaptation for division of labor, colony economics, or be nonadaptive. It was also suggested that perhaps this variation allows for niche specialization in workers foraging at different temperatures: larger bees might be better suited to forage at cooler temperatures and smaller bees might be better suited to forage at warmer temperatures. This we tested here using a large, enclosed growth chamber, where we were able to regulate the ambient temperature. We found no significant effect of ambient or nest temperature on the average size of bees flying to and foraging from a suspended feeder. Instead, bees of all sizes successfully flew and foraged between 16°C and 36°C. Thus, large bees foraged even at very hot temperatures, which we thought might cause overheating. Size variation therefore could not be explained in terms of niche specialization for foragers at different temperatures.

## 1. Introduction

Although a plant might be fertilized by a variety of organisms, bumble bees (*Bombus* spp.) possess many features to make them one of the most essential of crop pollinators [1–3]. Like some other bees, developing larvae are fed pollen as a protein source [4, 5], which necessitates their foragers visiting a large number of flowers to collect resources. Bumble bee foragers exchange information at flowers to improve foraging efficiency [6–10] and can also recruit nestmates to profitable types of food sources by transferring information about presence [11–14], quality [15, 16], and scent [14, 17] in the nest, although contrary to honey bees [18], the location of food sources is not communicated [14]. However, in contrast to many other bee species, bumble bees, with their larger size and plentiful insulation, are much hardier and faster pollinators [3, 19–21] and able to fly even in cold and wet conditions, down to temperatures of 5°C [22] or even lower (*Bombus polaris*, where workers are quite large, is capable of foraging at near freezing temperatures: [23–26]). Lastly, bumble bees are a relatively large genus compared to honey bees, thus providing many different types and sizes of foragers, able to handle a variety of floral styles and shapes [3, 27]. All of this results in bumble bees visiting many flowers, facilitating the effective transfer of pollen. However, despite their economic importance, bumble bees remain a relatively unstudied insect pollinator compared to honey bees.

**Figure 1:**
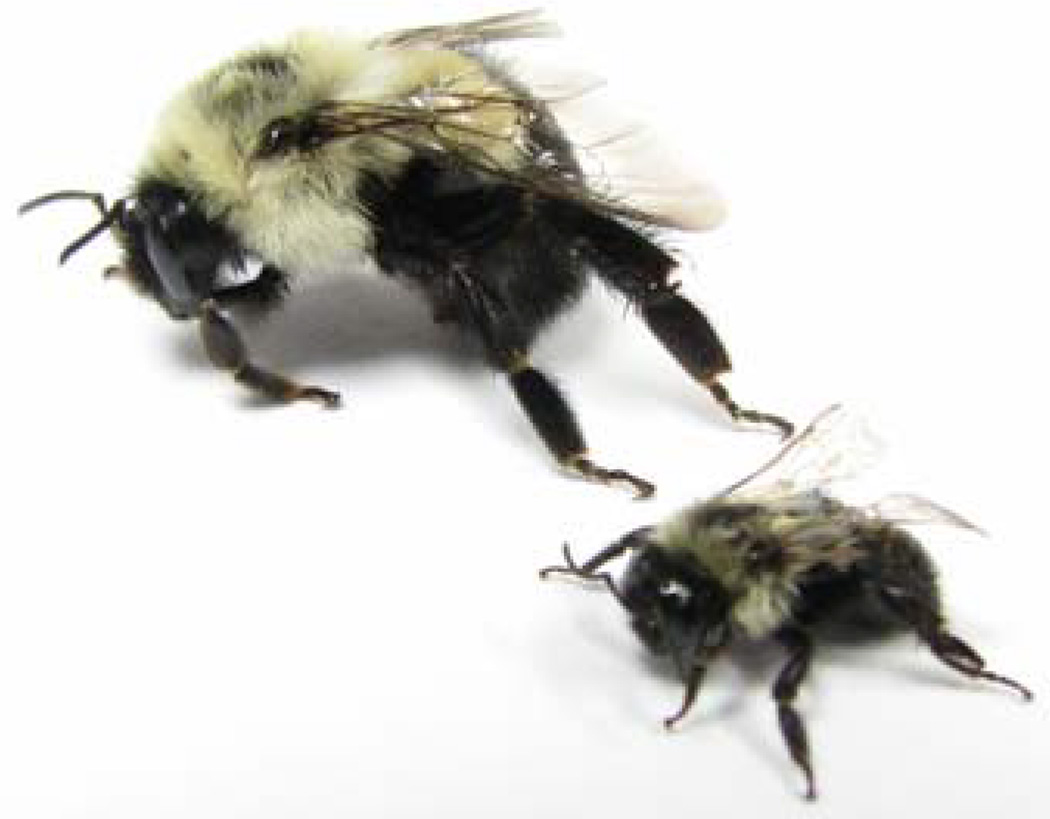
Bumble bees (here: *Bombus impatiens*) may display as much as 10-fold difference in mass between workers in the same nest, even though they are full sisters.

One important and unusual feature in bumble bees is that highly related worker sisters from the same colony will display as much as a 10-fold difference in mass (Figure 1; [3, 28]). Larger workers emerge from the center of the nest, where larvae receive more intense care due to high density of nurses [29, 30]. Body size predicts worker task allocation: larger bees tend to forage and smaller bees tend to be nurses [29, 31–33]. Is worker polymorphism therefore an adaptation for division of labor? Larger bees do perform better as foragers [34, 35], also reviewed in [32, 36]. However, specialization is generally weak in bumble bees [37], and it is not clear that small bees are particularly suited as nurses [38] (and Dornhaus, unpublished data). On the other hand, smaller workers may require less investment to produce and may be more robust to starvation [36]. The production of polymorphic workers may thus be a colony-level adaptation to increase colony efficiency or robustness. In addition, having workers of different sizes may also be an adaptation to foraging, akin to niche partitioning. For example, workers of different sizes may be ideally suited for flowers of differing corolla depths [39]. Alternatively, workers of different sizes may be suited to different temperatures at which the colony needs to forage. This is what we test here.

Bumble bees are cold-hardy foragers, especially compared to most bee species that are smaller. Their native range includes temperate, alpine, and even arctic zones [3, 40]. Nevertheless, thermoregulation is important even in most ectotherms [41], and a bumble bees’ flight muscles must be warmed to at least 30°C before flight is possible [3, 42–44]. Bumble bees, like some other insects, are capable of a type of endothermy that is achieved by rapid muscle contractions [19, 45–48]. Bumble bee body temperature may also be influenced by external factors like the ambient temperature and the quality of their food [42]. If ambient temperature is less than body temperature, the bumble bee will be susceptible to heat loss. Since body size affects the surface area-to-volume ratio, to which heat loss is related, this may prevent smaller bees from achieving flight temperature in colder weather [49–51]. On the other hand, while larger bees are better thermoregulators [52], they may be susceptible to overheating during flight [53]. This is because metabolic heat is not transferred to the environment as quickly in larger organisms. The maximum thoracic temperature that bumble bees can tolerate is 42°–44°C [43, 54]. The dramatic intracolony variation in worker body size may thus be linked to different bees’ abilities to forage at variable temperatures. If bumble bee size variation is an adaptation for foraging that allows for specialization, then larger workers should specialize in foraging at cooler temperatures and smaller workers should fly at hotter temperatures. Indeed, if workers from different bumble bee species are compared, those from colder climates are often larger [50]. However, in the same study, it was also shown that both “large” and “small” workers of *Bombus terrestris* (exact body sizes were not measured) could be collected in the field at all temperatures between 18° and 33°C [50].

Here we test whether ambient and nest temperatures determine which workers are allocated to foraging in the bumble bee (*Bombus impatiens*). To test this hypothesis, we systematically manipulated ambient temperatures in a large, enclosed flight chamber and observed marked foragers of known size who accessed a suspended feeder. We predicted that larger foragers would tend to forage in cooler temperatures and, conversely, smaller foragers would tend to forage in warmer temperatures.

## 2. Materials and Methods

### 2.1. Study Organism and Experimental Setup

We obtained 2 bumble bee colonies (*B. impatiens*; colonies 1 and 2) from Koppert Biological Systems (Romulus, MI). At the start of the experiment, colonies were queenright with typically 20–30 workers with brood; over the course of the experiment, colonies grew to a size of over 100 workers. We housed colonies in Plexiglas boxes (22 × 22 × 11 cm) with screened ventilation holes and an opening over the top through which we directly delivered pollen each day of the experiment. The nest boxes were placed inside a large drink cooler (61 × 33 × 37 cm) to simulate typical ground nesting (i.e., insulated) conditions. In this way, the nest box was kept in the dark; however, the foraging arena was on a 12 : 12 light : dark cycle. A petri dish of water, which we refilled daily, was placed inside the nest box as well. Each nest box was then connected to a separate foraging arena (58 × 36 × 40 cm) by plastic tubing. Inside the foraging arena, feeders were placed on platforms (8 × 8 × 10 cm) suspended from the mesh top of the arena, which required the bees to fly instead of walk to the food. We placed the entire experimental setup inside a growth chamber, which allowed us to regulate precisely the ambient temperature, while also measuring the nest temperature.

### 2.2. Data Collection

Before the experiment began, we marked a subset of worker bees by gluing unique number identification tags (“Opalithplättchen”) to their thorax. Although tagged workers were chosen at random, we pulled bees from the foraging area of the nest box to assure that tagged bees were potential foragers. We continued to tag bees throughout the experiment to maintain a population of tagged bees for observation. The tags did not interfere with normal bee behavior or flight. Data were collected 5 days a week. Each morning, we would make sure that at least 25% of the honeypots in the nest contained honey; this provided a standardization of worker motivation and recruitment [16]. If less than 25% of the pots were full, we would fill some of the pots with sugary solution (“BeeHappy”) by syringe. The growth chamber temperature was set to the experimental ambient temperature setting for that day at 9:00 hours. In this way, the chamber was heated or cooled to the specified temperature, which we verified by thermometers set both inside and outside the nest. Typically the nest box, insulated by the cooler, would not heat or cool as much as the room itself, which simulates natural conditions. The experimental ambient temperature was set between 16°C and 36°C. We randomized the order of experimental temperatures in 5°C increments (16, 21, 26, 31, 36°C). Colonies were monitored for stress at extreme high and low temperatures, even though the cooler provided a measure of insulation. We chose 36° as the maximum temperature because above it colonies showed signs of high stress, with many workers fanning and beginning to abandon brood. Below 16°C, very few or no bees foraged in our setup.

At 12:30, the chamber had always reached the desired temperature, and we placed feeders on the suspended platforms. Feeders were filled with sugary solution, which was always of the same concentration and quality (BeeHappy, Koppert Biological Systems, 1 : 1 diluted with water), as these factors influence the thoracic temperature of the bees [42]. We began data collection at 13:00. This allowed time for the bees to discover the food and initiate foraging [16]. For 90 minutes, we recorded the identity of any bee who successfully foraged (extended proboscis) at the feeders. Foraging at the suspended platforms required flight, which required sufficient heat with which to activate flight muscles. On 8 days, we additionally recorded for how many trips each foraging bee returned to the feeder.

At 14:30, we stopped data collection and fed each colony a teaspoon of pollen. Honeypots were verified as 25% full. The growth chamber temperature was set back to 26°C, and any dead bees were removed and stored in the freezer. After the experiment, we measured the thorax width of all the worker bees with digital calipers to the nearest 0.01 mm. Thorax width is a typical measurement of size in bumble bees [3].

## 3. Results

**Figure 2:**
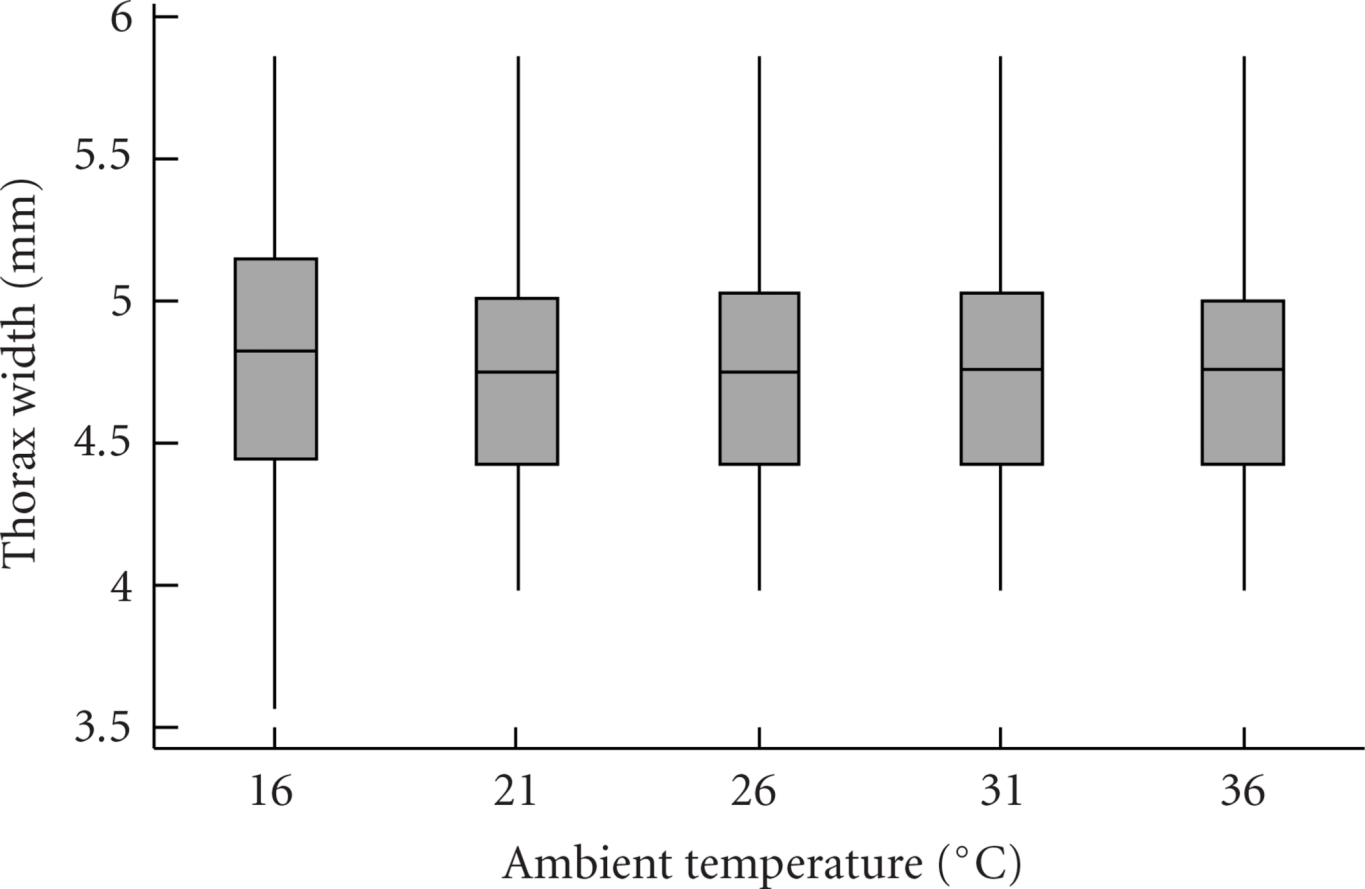
Bees of a wide range of body sizes were found to forage at all ambient air temperatures. Data are pooled here for both colonies; shown are medians (lines), quartiles (boxes), and ranges (whiskers) (*n* = 70 bees for 16°, *n* = 65 for 21°, *n* = 67 for 26°, *n* = 71 for 31°, *n* = 73 for 36°).

**Figure 3:**
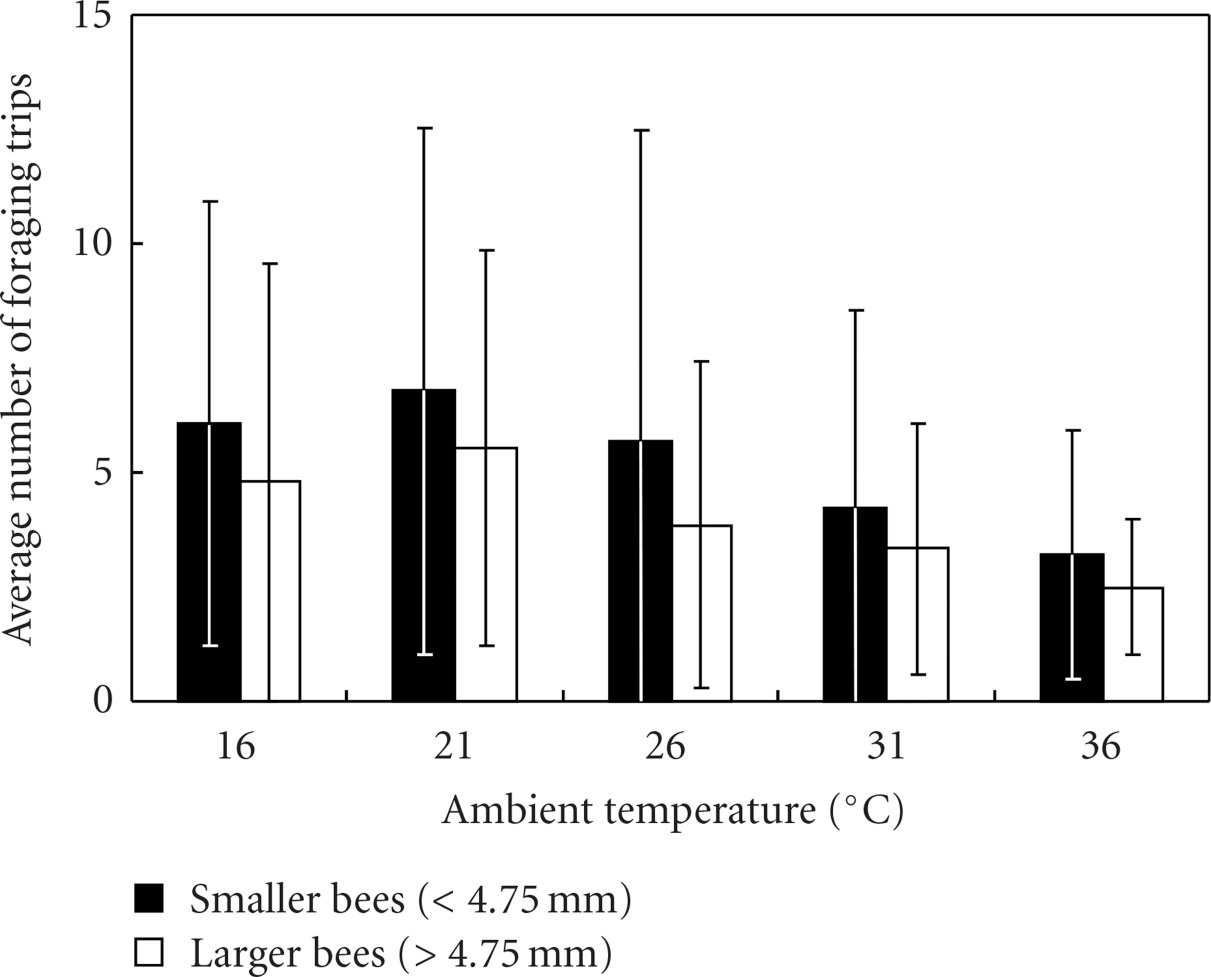
Foraging activity, measured as the number of trips per bee in one observation period, declined at higher temperatures. Shown is the average (with standard deviation) of all bees in the respective category across all days with the respective temperature (total *N* = 339 bees*days).

Overall, we found that all forager body sizes were measured at all temperatures (Figure 2). The number of trips made per observation period decreased at higher temperatures and was on average lower in larger foragers (defined here as foragers over 4.75 mm thorax width, Figure 3; ANOVA, df = 9, *R*^2^ = 0.86, ambient temperature *P* = .002, body size *P* = .028, interaction *P* = .61). It is not clear why larger bees made fewer trips; perhaps because they needed longer to fill their crop on each visit. This result is the same if, instead of the average number of trips across bees in the respective category, each bee’s number of trips is entered in the analysis separately (df = 338, *R*^2^ = 0.07, ambient temperature *P* = .004, body size *P* = .0002, interaction *P* = .43).

### 3.1. Worker Body Size Did Not Predict Average Temperature at Which She Foraged

**Figure 4:**
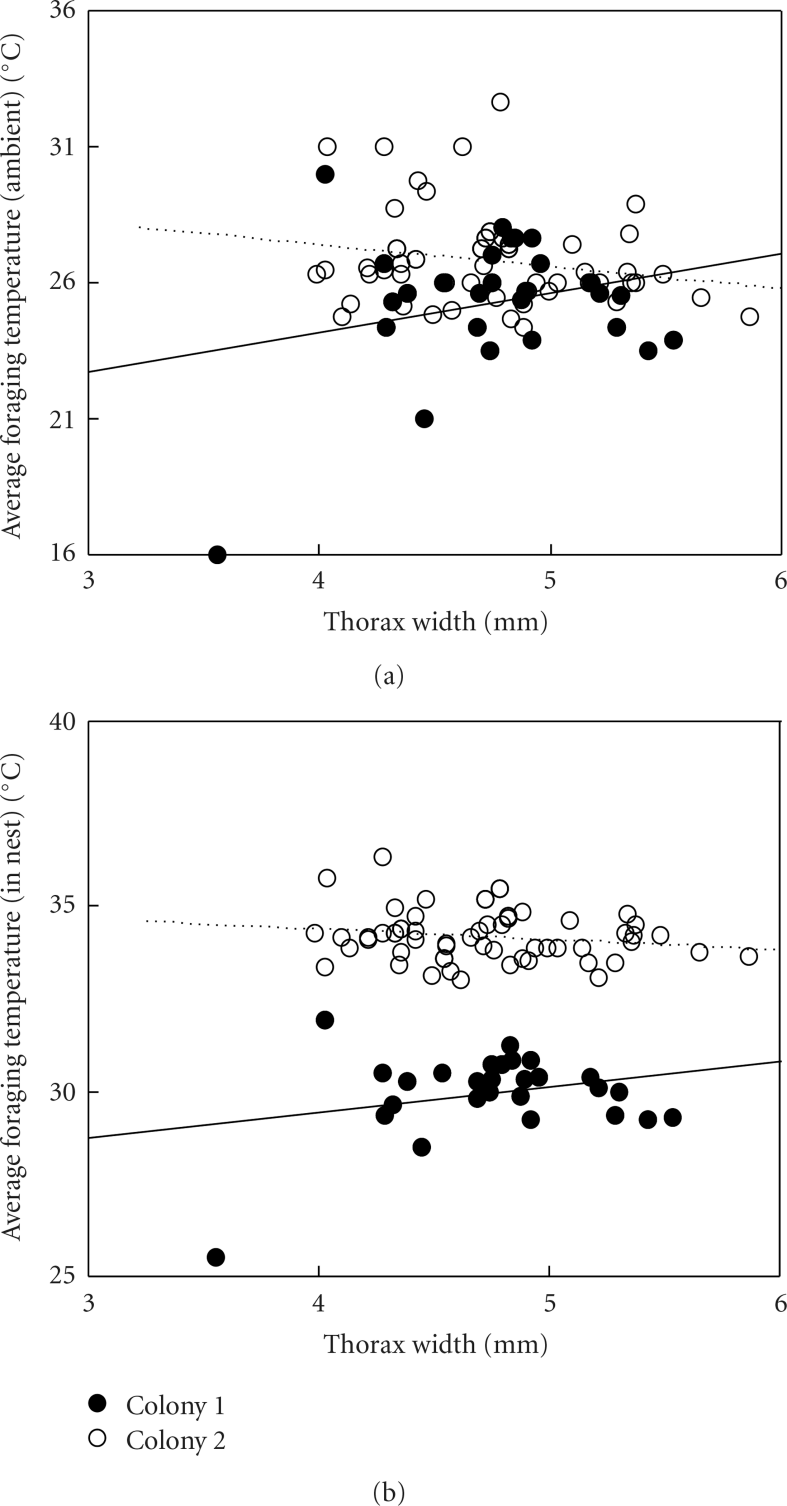
Foragers of different body sizes had similar maximum and minimum foraging temperatures within the temperature range studied here (up to 36°C ambient temperature). The relationship of thorax width and maximum foraging temperature was only significant if the outlier (marked with an arrow) of one bee which only made a single trip in the whole study was included. Shown are (a) ambient temperature and (b) temperature measured in nest; each data point is the maximum or minimum of all observations for that bee. In total 81 bees are shown.

Averaging the temperatures for the days on which each bee foraged, we found no effect of body size on foraging temperature, although there was a significant effect of colony as well as its interaction with body size (ANOVA, df = 80, *R*^2^ = 0.15, thorax width *P* = .57, colony *P* = .003, interaction *P* = .046; Figure 4(a)). Similarly, there was no relationship with average temperature measured in the nest when bees of different sizes foraged (*R*^2^ = 0.86, thorax width *P* = .35, colony *P* < .0001, interaction *P* = .035; Figure 4(b)).

### 3.2. Worker Body Size Did Not Correlate with Maximum Foraging Temperature

**Figure 5:**
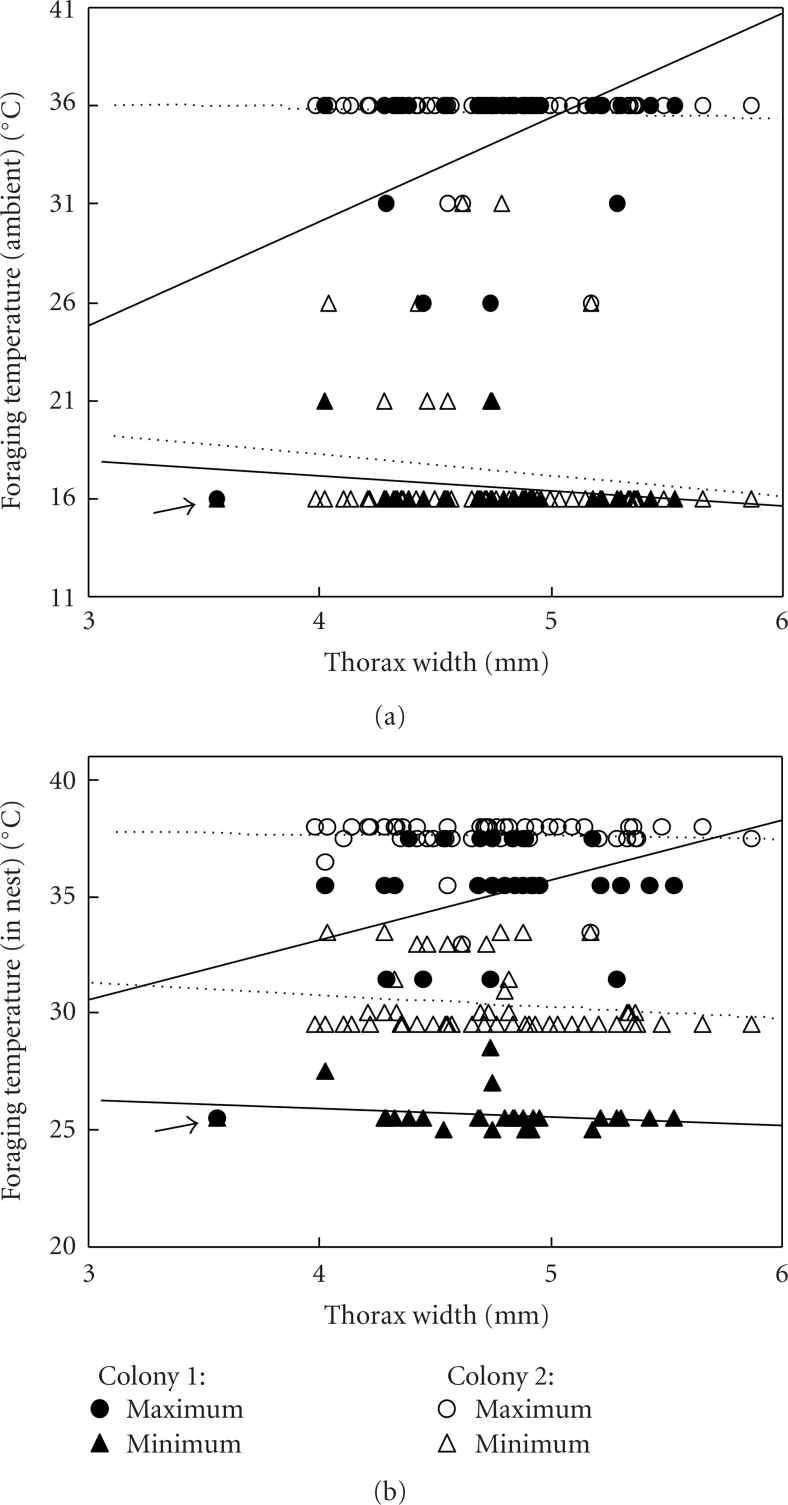
Foragers of different body sizes did not significantly differ in the average temperatures at which they foraged (although linear fits are shown, slopes are not significantly different from zero). However, colonies differed significantly from each other. Shown are (a) ambient temperature and (b) temperature measured in nest; each data point is the average temperature across all days on which that bee foraged (each bee foraged on average on 13.9 days), and in total, 81 bees are shown.

The maximum temperature, out of the temperatures tested by us, at which a worker would forage seemed at first predicted by body size, with larger bees foraging at higher maximal ambient temperatures (ANOVA, df = 80, *R*^2^ = 0.25, thorax width *P* = .0009, colony *P* = .016, interaction *P* = .0003; Figure 5(a)). However, there was the single outlier of one bee that was only seen foraging once, at 16°C (Figure 5(a)). Since this single trip entered the analysis as a maximum foraging temperature of 16°C, it strongly affected the results. If that bee is removed from the analysis of maximum foraging temperature, there is no remaining effect of body size (*R*^2^ = 0.25, thorax width *P* = .48, colony *P* = .11, interaction *P* = .26). The same was true for the relationship between body size and maximal in-nest temperature at which the bee foraged, although there was always an effect of colony on in-nest temperature (with the outlier: *R*^2^ = 0.42, thorax width *P* = .006, colony *P* < .0001, interaction *P* = .003; without the outlier: *R*^2^ = 0.37, thorax width *P* = .80, colony *P* < .0001, interaction *P* = .64; Figure 5(b)).

### 3.3. Worker Body Size Did Not Correlate With Minimum Foraging Temperature

Neither minimum ambient nor minimum in-nest temperature at which a worker foraged was predicted by its body size (ANOVA, df = 80, ambient: *R*^2^ = 0.03, thorax width *P* = .29, colony *P* = .28, interaction *P* = .85; in-nest: *R*^2^ = 0.77, thorax width *P* = .21, colony *P* < .0001, interaction *P* = .80; Figures 5(a) and 5(b)).

## 4. Discussion

We found no significant effect of ambient or nest temperature on the average size of foragers flying to a suspended feeder. Instead, bees of all sizes successfully flew and foraged at ambient temperatures between 16°C and 36°C. These results lead us to reject the hypothesis that producing small workers may be a colony-level adaptation to foraging at warmer temperatures in bumble bees.

Larger animals are often thought to be more prone to overheating, because of their smaller surface to volume ratio; on the other hand, smaller animals may suffer detrimental heat loss. This temperature-body size relationship is thought to pose constraints on the evolution of very large animals, such as dinosaurs [55, 56], but it also is thought to affect distribution and evolution of a variety of other taxa (e.g., mammals: [57]; birds: [58]; reptiles: [59]; insects: [41, 60, 61]). In dinosaurs specifically, larger body size was likely associated with reduction in loss of metabolic heat as well as heat from solar radiation to such a degree that overheating became a risk and increased blood flow to the skin and other adaptations to increase heat loss became necessary [56, 59], although this may apply strongly at body sizes of over 10 kg [56]. As a consequence of this, it has been hypothesized that larger species tend to be found in cooler climates, and this relationship is known as “Bergmann’s rule” [60, 62–66]. Species may also evolve different body sizes in response to climatic change [57]. However, evidence for “Bergmann’s rule” remains contradictory [67, 68]. Body size-temperature relationships are idiosyncratic in different taxa and may be caused by indirect effects, such as precipitation or seasonality correlating with temperature, and affecting body size across taxa [57]. In addition, developmental regulation of body size can be complex and vary even among related taxa [69]. Also, body cooling by wind can significantly increase heat loss [56], an effect that should be even more relevant in flying bees.

We were curious to see whether thermal ecology would also affect the evolution of size polymorphism within a species, namely, among workers of bumble bee colonies. Across species, thermal ecology may result in niche separation: for example, the ability of many bumble bee species (*B. terrestris*, *B. pascuorum*, and *B. hortorum*) to fly at much cooler temperatures than honey bees can result in temporal separation between the two families [22]; thermal niches may also exist among ant species [70, 71]. Within individual bumble bee colonies, larger bees were proposed to fly at lower temperatures, so they would be expected to fly earlier and later in the day and on colder days, whereas smaller bees might have been more resistant to overheating, therefore flying at midday and on warmer days [3]. This idea that bumble bee worker body size may predict foraging temperature has been proposed a number of times [3, 47, 72]. However, while it is still possible that only larger bees can forage at extremely low temperatures (<16°C), our study shows that small bees do not have a higher maximal temperature tolerance, as bees of all sizes still forage at >36°C. This result is consistent with as study by Peat et al., who also found no evidence that ambient temperature affected the activity of workers of different sizes [50].

In summary, it is likely that overheating does not constrain foraging activity for large bumble bees as long as outside temperatures remain within the tolerable limits. Flying bees may not overheat easily because of their overall small size, cooling effects of air movement while flying, and distance to the ground. It is also possible that bee would have been more susceptible to overheating had they been forced to fly longer distances than in our study. In future studies, it would be interesting to see whether flight distance affects forager susceptibility to overheating, and also whether individual experience will affect the temperatures at which bumble bee workers decide to forage. We also found significant colony differences in the average temperature at which workers foraged. There may thus be colony variation in worker temperature preferences or in how well colonies regulate in-nest temperature. However, there was no significant interaction between colony and the body size-maximum/minimum foraging temperature, indicating that in neither colony large and small workers differed in the range of temperatures at which they foraged.

In our experiment, foraging activity decreased at the highest temperatures but had not yet completely ceased, even when nest temperatures reached >38°C. At these temperatures, many bees are fanning the brood in the nest to cool the developing larvae, which may have lower heat tolerance [73–78]. Foraging activity may thus have decreased because foragers were occupied with nest thermoregulation more than because they were unable to fly at high outside temperatures. The fact that temperatures in the nest reached higher values than those outside opens up other interesting questions: clearly overheating and lack of effective shedding of metabolic heat may not be problematic at the individual level, but may be problematic at the colony level in spite of behaviors that regulate nest temperature in bumble bees [79–88]. It would be interesting for future research to compare the ventilation structures and other thermal adaptations of nests of tropical and temperate bumble bees and of larger and smaller colonies (as in other social insects: [89–91]).

Our study is one of a growing list of studies showing that in bumble bees, larger workers outperform smaller workers at many tasks, or perform equally well [32, 34, 35, 38], although see [36, 39] for how smaller workers might possess adaptive advantages. It is thus possible that small workers, rather than being adapted to particular conditions or tasks, are produced because they are less costly (both in production and maintenance), yielding a better gain per investment for some tasks compared to larger workers.
